# *TMEM173* rs7447927 genetic polymorphism and susceptibility to severe enterovirus 71 infection in Chinese children

**DOI:** 10.1002/iid3.742

**Published:** 2022-11-25

**Authors:** Jie Song, Yedan Liu, Ya Guo, Zhenghai Qu, Peipei Liu, Fei Li, Chengqing Yang, Fan Fan, Zongbo Chen

**Affiliations:** ^1^ Department of Pediatrics The Affiliated Hospital of Qingdao University Qingdao Shandong China; ^2^ Department of Pediatrics Shandong Provincial Hospital Affiliated to Shandong First Medical University Jinan Shandong China; ^3^ Department of Pharmacology and Toxicology University of Mississippi Medical Center Jackson Mississippi USA

**Keywords:** TMEM173, single‐nucleotide polymorphism, IFN‐α, EV71

## Abstract

**Introduction:**

This study was designed to explore the association between the *TMEM173* polymorphism (rs7447927) and the severity of enterovirus 71 (EV71) infection among Chinese children.

**Methods:**

The *TMEM173* polymorphism was identified in EV71‐infected patients (*n* = 497) and healthy controls (*n* = 535) using the improved multiplex ligation detection reaction (iMLDR). The interferon‐α (IFN‐α) serum levels were detected using enzyme linked immunosorbent assay (ELISA).

**Results:**

The frequencies of the GG genotype and G allele of *TMEM173* rs7447927 in the mild EV71 infection and severe EV71 infection groups were markedly higher than those in the control group. The GG genotype and G allele frequencies in severely infected EV71 patients were significantly higher than those in mildly infected EV71 patients. Severely infected EV71 patients with the GG genotype had higher white blood cell counts (WBC), and C‐reactive proteins (CRP), and blood glucose (BG) levels, longer fever duration, higher vomiting frequency, spirit changes, and electroencephalography (EEG) abnormalities. IFN‐α serum concentration in severely infected patients was significantly higher than in the mildly infected group. The IFN‐α concentration in the GG genotype was significantly higher compared with those in the GC and CC genotypes in severe cases.

**Conclusions:**

The *TMEM173* rs7447927 polymorphism was associated with EV71 infection susceptibility and severity. The G allele and GG genotype are susceptibility factors in the development of severe EV71 infection in Chinese children.

## Graphical abstract

1. The TMEM173 rs7447927 polymorphism was associated with susceptibility and severity of EV71 infection. The G allele and GG genotype are susceptibility factors in the development of severe EV71 infection in Chinese children. 2. Severely infected EV71 patients with the GG genotype had higher white blood cells (WBC), C‐reactive proteins (CRP), and BG levels, a longer duration of fever, and a higher frequency of vomiting, spirit changes, and electroencephalography (EEG) abnormalities. 3. The serum concentration of interferon‐α (IFN‐α) in severely infected patients was significantly higher than that in the mildly infected group. The IFN‐α concentration in the GG genotype was significantly higher than that in the GC and CC genotypes in severe cases.

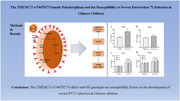

## 1 INTRODUCTION

Enterovirus 71 (EV71), a single positive‐sense‐strand, neurovirulent RNA virus, is a well‐known pathogen that causes hand‐foot‐and‐mouth disease (HFMD).^1^, ^2^ Most children infected with EV71 manifest only mild symptoms (fever and rashes on the mouth, hands, feet, and hip); however, a few children have symptoms that are accompanied by serious central nervous system complications such as aseptic meningitis, encephalomyelitis, brainstem encephalitis, flaccid paralysis, and neurogenic pulmonary edema.^3^ In 2008, a large‐scale Asia‐Pacific pandemic in China led to the infection of 490,000 children with EV71, where of 126 patients died.^4^ The clinical manifestations of the disease in infected children differ, depending on virulence and host immunity. Recent studies have mainly focused on exploring the relationship between EV71 infection and host immunity at the molecular level, and it has been found that human gene polymorphisms, such as those of *CPT2*, *OAS2*, and *IL‐17F*, are related to infection susceptibility and severity.^5^, ^6^, ^7^, ^8^, ^9^

The transmembrane protein 173 (TMEM173), also known as MPYS, mediates the interferon regulatory factor 3 (IRF3) activation (MITA), endoplasmic reticulum interferon stimulator (ERIS), stimulator of interferon genes (STING), and stimulator of interferon response cyclic guanosine monophosphate‐adenosine monophosphate (cGAMP) interactor 1 (STING1). Human *TMEM173* is located on chromosome 5q31.2, contains eight exons, and encodes a transmembrane protein between the endoplasmic reticulum and Golgi in response to the presence of cytosolic dsDNA as well as an adaptor protein that mediates the production of type I interferon.^10^ *TMEM173* plays an important role in the cross‐reaction between innate immunity, inflammation, autophagy, and cell death in response to invasive microbial pathogens or endogenous host damage.^11^ Studies have shown that *TMEM173* is involved in the disease progression of multiple viruses that infect the human body, but the relationship between EV71 and *TMEM173* is not clear. *TMEM173* gene has rs7447927, rs13166214, rs55792153, and other single nucleotide polymorphisms (SNP) loci, but only the rs7447927 locus has been reported to be associated with oesophageal cancer, dose‐related telomere damage, and other diseases. Therefore, this study aimed to explore the relationship between the *TMEM173* rs7447927 locus and EV71 infection susceptibility and severity.

## 2 MATERIALS AND METHODS

### 2.1 Case selection

**Figure 1:**
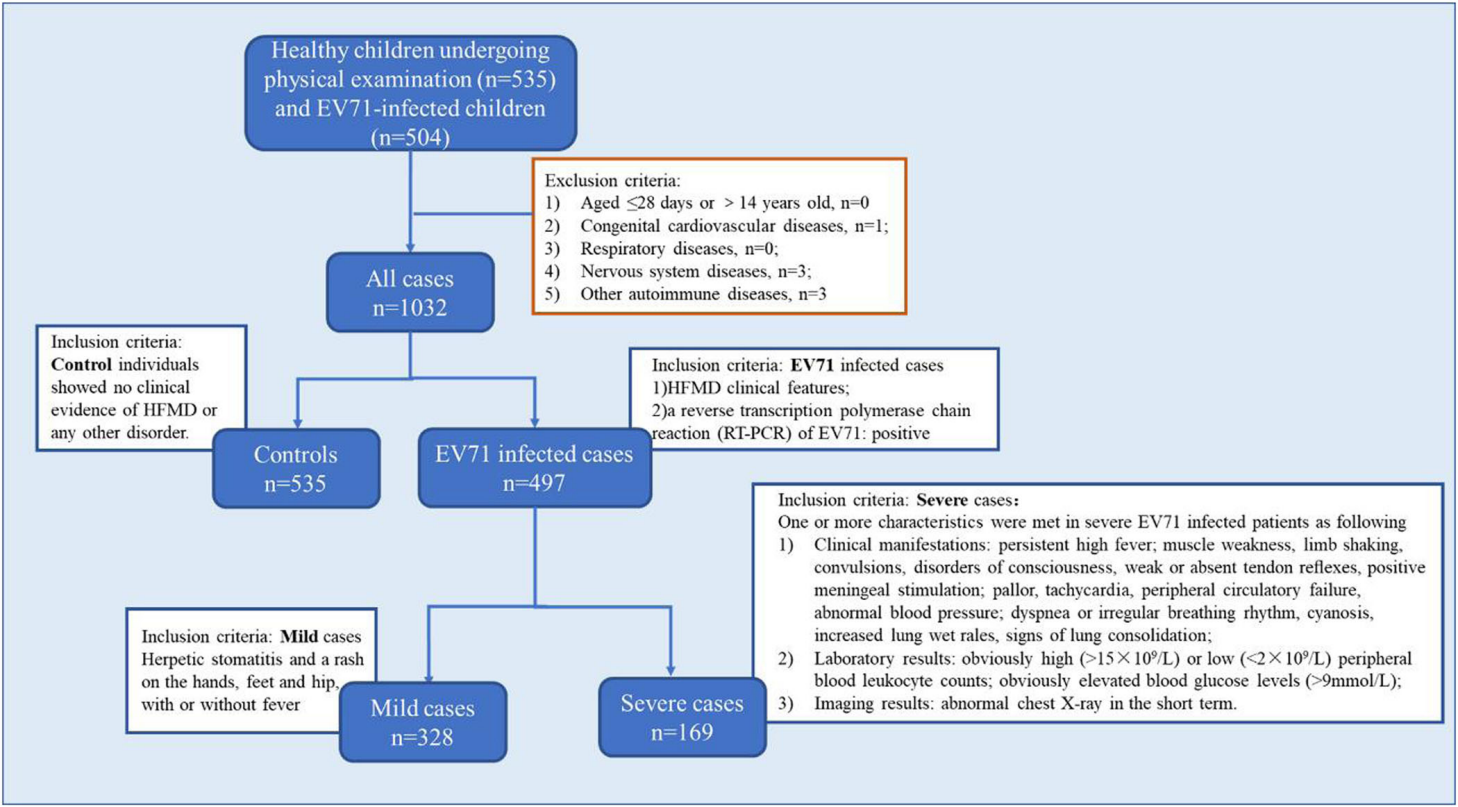
Flowchart demonstrating the patients included in the study as well as the inclusion and exclusion criteria

We examined 535 healthy children undergoing physical examination and 504 EV71‐infected children in the Affiliated Hospital of Qingdao University, Qingdao Women & Children's Hospital, and Affiliated Hospital of Jining Medical University between 2013 and 2019. Seven cases in the infected group were excluded due to underlying diseases: congenital heart disease (*n* = 1), epilepsy (*n* = 3), and other immune system diseases (*n* = 3). EV71 infection was confirmed by both an analysis of the clinical features and a reverse transcription polymerase chain reaction (RT‐PCR). RNA was extracted from stool specimens obtained from the patients on the day after admission. Clinical and laboratory data were collected. Inclusion/exclusion criteria in our study are shown in Figure 1. The severity of EV71 infection was clarified according to the guidelines of HFMD diagnosis and treatment (2010) by the Chinese Ministry of Health.^12^

### 2.2 Genomic DNA isolation and genotyping of *TMEM173 rs7447927*

A commercial kit (Qiagen) was used to extract genomic DNA from peripheral blood. An iMLDR technique impoldered by Genesky Biotechnologies Inc. was used to detect the *TMEM173* rs7447927 polymorphism genotype. The product was 201 bp in size. For rs7447927 gene polymorphism analysis, PCR was performed using the forward primer 5ʹ‐CAGGGCTAGGCATCAAGGGAGT−3ʹ and reverse primer 5ʹ‐ GACCCTTTGGGGGCTAGGAGAG −3ʹ. The PCR procedure was as follows: 95°C for 2 min; 11 cycles at 94°C for 20 s, 65–0.5°C/cycle for 40 s; 72°C for 90 s; 24 cycles at 94°C for 20 s, 59°C for 30 s; 72°C for 90 s; followed by 72°C for 2 min and held at 4°C. The PCR products were purified by digestion with 1U shrimp alkaline phosphatase at 37°C for 1 h and at 75°C for 15 min. The ligation reaction mixture contained 10× ligase buffer 2 μl, Taq DNA ligase 0.2 μl, probe mixture 1 μl, and the purified PCR product mixture 3 μl. In a double connection reaction, each site contained two 5′ ends of allele‐specific probes, followed by the 3′ end of an allele‐specific probe. Each allele‐specific interlinkage product was distinguished by its immune fluorescence, but different loci were distinguished by the different lengths added to the 3′ end of the allele‐specific probe. The probes used were as follows:

rs1946519FC: TCTCTCGGGTCAATTCGTCCTTGCAGGGAGGCTAGGTGGTG, rs1946519FG: TGTTTGGGCCGGTTAGTGCAGGGAGGCTAGGTGGTGG, and rs1946519FP: ACCAGGTACCGGAGAGTGTGCTTTTTTTTTTTTTTTTTTTTTTTTTTTTTTTTTTTTT.

The ligation cycling step consisted of 35 cycles at 94°C for 1 min, 56°C for 4 min, followed by holding at 4°C. Alleles were genotyped using a 3130xl Genetic Analyzer (ABI), and the raw data were analysed using Gene Mapper 4.0 (Applied Biosystems) (Table 1).

**Table 1:** Primers and PCR conditions

Item		Sequence (5′−3′)	The PCR conditions
TMEM173 rs7447927	Forward primer	CAGGGCTAGGCATCAAGGGAGT	95°C for 2 min; 11 cycles at 94°C for 20 s, 65°C–0.5°C/cycle for 40 s, and 72°C for 1 min 30 s; and 24 cycles at 94°C for 20 s, 59°C for 30 s, and 72°C for 1 min 30 s, followed by 72°C for 2 min and holding at 4°C.
	Reverse primer	GACCCTTTGGGGGCTAGGAGAG	
TMEM173 rs7447927probes	FC	TCTCTCGGGTCAATTCGTCCTTGCAGGGAGGCTAGGTGGTGC	38 cycles at 94°C for 1 min and 56°C for 4 min, and then holding at 4°C
	FG	TGTTCGTGGGCCGGATTAGTGCAGGGAGGCTAGGTGGTGG	
	FP	ACCAGGTACCGGAGAGTGTGCTTTTTTTTTTTTTTTTTTTTTTTTTTTTTTTTTTTTT	
EV71 VP1	EV71‐S	GTTCTTAACTCACATAGCA	95°C for 15 min, followed by 40 cycles of 95°C for 10 s, and 60°C for 60 s
	EV71‐A	TTGCAAAAACTGAGGGTT	

### 2.3 Detection of EV71 loading

Peripheral blood lymphocytes were extracted according to the manufacturer's instructions (Solarbio) on the second day after admission. Total RNA was purified by TRIzol reagent (Invitrogen) in accordance with the manufacturer's protocol, and the concentration and quality of the RNA were assessed by A QuickDrop spectrophotometer. The SYBR‑Green Real‑Time PCR kit (Vazyme) was used to reverse‐transcribe the mRNA into complementary DNA (cDNA). A standard curve for calculating the copy numbers of viral RNA was built by a series of dilutions (1 × 107, 1 × 106, 1 × 105, and 1 × 104 copies/μl of a DNA fragment) in various samples. The ligation cycling step consisted of 95°C for 15 min and 40 cycles of thermal cycling at 95°C for 10 s and 60°C for 60 s. Quantitative RT‐PCR was performed using a Linegene9660 system (Thermo Fisher Scientific). The specific primers used were as follows: EV71‐S: GTTCTTAACTCACATAGCA, EV71‐A: TTGCAAAAACTGAGGGTT (Table 1).

### 2.4 Estimation of interferon‐α (IFN‐α) levels

Plasma IFN‐α levels were detected using ELISA kits (Thermo Fisher) in 99 controls, 73 mild EV71‐infected cases, and 60 severe EV71‐infected cases. The sensitivity of IFN‐α detection was 3.2 pg/ml. Each sample was repeated three times, and these values were in the linear portion of the standard curve.

### 2.5 Statistical analysis

A Hardy–Weinberg equilibrium (HWE) was used to test the proportion of genotypes in controls. The frequencies of genotypes and alleles in different groups were compared using the *χ* ^2^ test. The relationships between rs7447927 polymorphism and EV71 susceptibility and severity of infection were evaluated by calculating the odds ratio (OR) and 95% confidence intervals (95% CI) using logistic regression. Parameters of normal distribution were expressed as means ± standard deviation (SD) and analysed via one‐way analysis of variance (ANOVA), followed by Student's *t* test. Nonparametric data were analysed using the Kruskal–Wallis test and expressed as median values (25th–75th percentile). All statistical analyses were performed using SPSS 21.0 (IBM SPSS software), and significance was set at *p* < .05.

## 3 RESULTS

### 3.1 Study population

A total of 535 controls (ages 5.16 [2.79–8.00] years, 268 males) and 504 EV71‐infected patients (ages 5.77 [3.62–7.84] years, 247 males) were examined. There were no significant differences in age (Z = 1.840, *p* = .066) or gender (χ^2^ = 0.001, *p* = .970) between EV71‐infected patients and controls. The EV71‐infected patients were divided into two groups: 169 severely infected patients (ages 5.66 [3.50–7.51] years, 89 males) and 328 mildly infected patients (ages 5.77 [3.62–7.84] years, 158 males). No significant differences in age (Z = 0.417, *p* = .677) or gender (*χ* ^2^ = 0.900, *p* = .343) were observed between patients with severe or mild EV71 infections.

### 3.2 Distribution of genotypes and alleles of *TMEM173*

The genotypic and allelic distributions of each group obeyed Hardy–Weinberg equilibrium (*p* > .05). EV71‐infected patients had a significantly higher frequency of the GG (*p* < .001), GG + GC genotypes (*p* = .001), and G allele (*p* = .001), than the controls (Table 2). The same result was obtained when comparing severely infected EV71 patients with mildly infected patients (*p* < .001, *p* < .001, and *p* = .001, respectively) (Table 3).

**Table 2:** Genotype and allele frequencies of the TMEM173 rs7447927 polymorphism in EV71‐infected patients and controls

TMEM173 rs7447927	EV71‐infected patients (*n* = 497)	Controls (*n* = 535)	*χ* ^2^	*p* value	OR (95%CI)
Genotype					
GG	133 (26.76%)	123 (22.99%)			
GC	261 (52.52%)	242 (45.23%)			
CC	103 (20.72%)	170 (31.78%)	16.174	<.001	
GG + GC	394 (79.28%)	365 (68.22%)			
CC	103 (20.72%)	170 (31.78%)	16.174	.001	2.302 (1.469–3.607)
Allele					
G	527 (53.02%)	488 (45.61%)			
C	467 (46.98%)	582 (54.39%)	11.323	.001	1.346 (1.132–1.600)

Abbreviations: CI, confidence interval; OR, odds ratio.

**Table 3:** Genotype and allele frequencies of the TMEM173 rs7447927 polymorphism in mild and severe EV71‐infected cases

TMEM173 rs7447927	Severe EV71 patients (*n* = 169)	Mild EV71 patients (*n* = 328)	*χ* ^2^	*p* value	OR (95% CI)
Genotype					
GG	51 (30.18%)	82 (25.00%)			
GC	101 (59.76%)	160 (48.78%)			
CC	17 (10.06%)	86 (26.22%)	17.734	<.001	
GG + GC	152 (89.94%)	242 (73.78%)			
CC	17 (10.06%)	86 (26.22%)	17.729	<.001	3.177 (1.818–5.554)
Allele					
G	203 (60.06%)	324 (49.39%)			
C	135 (39.94%)	332 (50.61%)	10.193	=.001	1.541 (1.181–2.011)

Abbreviations: CI, confidence interval; OR, odds ratio.

### 3.3 Analysis of clinical features

Among EV71‐infected patients, those with the GG genotype had higher WBC (*p* < .001), C‐reactive proteins (CRP) (*p* < .001), and longer fever duration (*p* = .002). There were no significant differences in the serum levels of alanine aminotransferase (ALT), aspartate aminotransferase (AST), cardiac creatine kinase‐MB fraction (CK‐MB), or BG among the different genotypes. There were no significant differences in the frequencies of vomiting, seizure, spirit change, brain magnetic resonance imaging (MRI) abnormalities, or abnormal electroencephalography (EEG) frequencies among children with GG, GC, and CC genotypes (Table 4). In severely infected cases, children with the GG genotype had higher WBC (*p* < .001), CRP (*p* < .001), ALT (*p* = .049), AST (*p* = .041), BG (*p* < .001) levels and longer fever duration (*p* < .001). In addition, the percentages of vomiting (*p* = .017), spirit change (*p* = .045), and EEG abnormalities (*p* = .041) in patients with the GG genotype were significantly higher than those in patients with the other two genotypes. There were no significant differences in gender, age, and EV71 load among the different genotypes in both the mild and severe infection groups (Table 5).

**Table 4:** Characteristic of EV71‐infected group according to different genotypes

Parameters	GG (*n* = 133)	GC (*n* = 261)	CC (*n* = 103)	χ^2^/F/Z	*p* value
Male (n = )	66	123	58		
Female (n = )	67	138	45	2.492	.288^a^
Age (years)	5.45 ± 2.78	6.16 ± 2.76	5.81 ± 3.30	1.790	.168^b^
Duration of fever (days)	3.00 (2.00–5.00)	3.00 (2.00‐4.00)	3.00 (2.00‐4.00)	12.655	=.002^c^
WBC (×10^9^/L)	10.32 ± 2.54	7.40 ± 2.22	7.42 ± 2.31	77.466	<.001^b^
CRP (mg/L)	8.74 ± 2.49	6.52 ± 1.93	6.26 ± 1.32	67.768	<.001^b^
ALT (U/L)	38.11 (30.28–48.60)	37.00 (28.26–46.60)	35.10 (27.54–42.40)	4.290	.117^c^
AST (U/L)	39.64 (29.19–56.60)	38.10 (30.36–58.70)	37.79 (28.16–46.45)	3.333	.189^c^
CK‐MB (U/L)	30.71 (19.50–39.21)	28.10 (20.68–40.13)	26.40 (18.41–39.88)	0.756	.685^c^
BG (mmol/L)	6.93 ± 1.59	6.98 ± 1.20	6.73 ± 1.15	1.179	.308^b^
Vomiting	58 (43.60%)	111 (42.50%)	43 (41.70%)	0.086	.958^a^
Seizure	64 (48.12%)	103 (39.46%)	47 (45.63%)	2.873	.238^a^
Spirit change	75 (56.39%)	117 (44.83%)	45 (43.69%)	5.554	.062^a^
Brain MRI Abnormal	53 (39.85%)	101 (38.70%)	35 (33.98%)	0.953	.621^a^
EEG Abnormal	65 (48.87%)	124 (47.51%)	49 (47.57%)	0.071	.965^a^
EV71 load (log10 copies/μl)	5.33 ± 1.25	5.46 ± 0.98	5.34 ± 1.31	0.718	.488^b^

Abbreviations: ALT, alanine aminotransferase; AST, aspartate aminotransferase; BG, blood glucose; CRP, C‐reactive protein; CK‐MB, cardiac creatine kinase‐MB fraction; EEG, electroencephalography; MRI, Magnetic Resonance Imaging; WBC, white blood cell count.

^a^ Groups compared using 2 × 3 *χ* ^2^ test.

^b^ Groups compared using the ANOVA, values expressed as mean ± SD.

^c^ Groups compared using the Wilcoxon rank‐sum test, values expressed as median (25th–75th percentile values).

**Table 5:** Characteristic of severe EV71 infection group according to different genotypes

Parameters	GG (*n* = 51)	GC (*n* = 101)	CC (*n* = 17)	*χ* ^2^/t/*Z*	*p* value
Male (n = )	26	55	9		
Female (n = )	25	46	8	0.165	.921^a^
Age (years)	5.59 ± 2.96	5.745 ± 2.95	5.472 ± 1.92	0.153	.858^b^
Duration of fever (days)	6.00 (5.00–7.00)	5.00 (4.00‐7.00)	4.50 (2.75–5.50)	18.36	<.001^c^
WBC (×10^9^/L)	10.49 ± 2.85	7.85 ± 2.49	7.59 ± 3.12	17.920	<.001^b^
CRP (mg/L)	8.54 ± 2.92	6.95 ± 2.36	6.41 ± 1.75	8.318	<.001^b^
ALT (U/L)	43.80 (34.55–54.30)	37.40 (26.50‐48.20)	37.30 (30.30–45.40)	6.016	.049^c^
AST (U/L)	65.20 (36.95–81.20)	58.70 (34.80‐74.30)	49.10 (27.60–55.90)	6.401	.041^c^
CK‐MB (U/L)	34.10 (17.70–45.45)	30.00 (19.25‐44.50)	30.60 (16.20–41.40)	0.352	.839^c^
BG (mmol/L)	8.69 ± 2.49	7.09 ± 1.53	6.23 ± 1.64	16.504	<.001^b^
Vomiting	35 (68.63%)	46 (45.54%)	7 (41.18%)	8.134	.017^a^
Seizure	31 (60.78%)	57 (56.44%)	9 (52.94%)	0.416	.812^a^
Spirit change	36 (70.59%)	51 (50.50%)	8 (47.06%)	6.202	.045^a^
Brain MRI Abnormal	25 (49.02%)	40 (39.60%)	6 (35.29%)	1.583	.453^a^
EEG Abnormal	34 (66.67%)	47 (46.53%)	7 (41.18%)	6.402	.041^a^
EV71 load (log10 copies/μl)	5.26 ± 1.47	5.34 ± 0.94	5.39 ± 1.16	0.116	.890^b^

Abbreviations: ALT, alanine aminotransferase; AST, aspartate aminotransferase; BG, blood glucose; CRP,C‐reactive protein; CK‐MB, cardiac creatine kinase‐MB fraction; EEG, electroencephalography; MRI, Magnetic Resonance Imaging; WBC, white blood cell count.

^a^ Groups compared using *χ* ^2^ test.

^b^ Groups compared using the ANOVA, values expressed as mean ± SD.

^c^ Groups compared using the Wilcoxon rank‐sum test, values expressed as median (25th–75th percentile values).

### 3.4 Correlation between the *TMEM173 rs7447927* polymorphism and serum IFN‐a level

**Figure 2:**
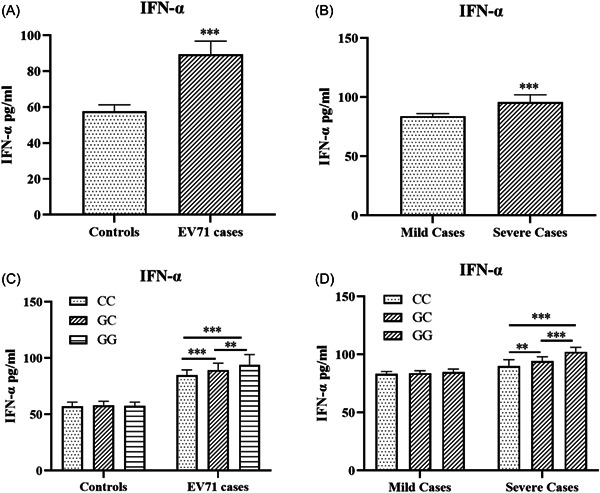
Level of interferon‐α (IFN‐α) in peripheral blood lymphocytes were detected in 99 controls, 73 mild EV71‐infected cases, and 60 severe EV71‐infected cases. Values expressed as mean ± SD. (A) The level of IFN‐α in peripheral blood lymphocytes in EV71‐infected cases was significantly higher than in controls (*p* ＜  0.001). (B) The level of IFN‐α in peripheral blood lymphocytes in severe EV71‐infected cases was significantly higher than in mild cases (*p* ＜ 0.001). (C). The level of IFN‐α in peripheral blood lymphocytes in GG genotypes in EV71 patients were significantly higher than in GC and CC genotypes patients (*p* < .01 and *p* < .001, respectively), but there was no difference in controls. (D). The level of IFN‐α in peripheral blood lymphocytes in GG genotypes in severe EV71‐infected patients were significantly higher than in GC and CC genotypes patients (*p* < .001 and *p* < .001, respectively), but there was no difference in mild cases. **p < .01, ***p < .001.

IFN‐α serum levels were noticeably higher in EV71‐infected patients (89.34 ± 7.45 pg/ml, *p* < .001) compared with those in uninfected children (57.69 ± 3.57 pg/ml) (Figure 2A). IFN‐α concentrations were significantly higher in severely infected cases (95.92 ± 6.13 pg/ml, *p* < .001) compared with those in mildly infected cases (83.92 ± 2.32 pg/ml) (Figure 2B). In the infected group, the serum concentration of IFN‐α in patients with the GG genotype (93.88 ± 9.28 pg/ml, *p* = .001 and *p* < .001, respectively) was significantly higher than those in patients with GC (89.38 ± 6.07 pg/ml) and CC (85.08 ± 4.47 pg/ml) genotypes, but there was no obvious difference among the genotypes in controls (Figure 2C). In severely infected patients, the serum level of IFN‐α in patients with the GG genotype (102.31 ± 3.84 pg/ml, *p* < .001 and *p* < .001, respectively) was significantly higher than in patients with the GC (94.20 ± 3.90 pg/ml) and CC genotypes (89.93 ± 5.58 pg/ml), but there was no significant difference among the genotypes in mildly infected patients (Figure 2D).

## 4 DISCUSSION

Severe EV71 infections are caused by a virus that threatens the safety of children. Although major breakthroughs have been made in EV71‐related vaccine research, targeted treatment measures are currently lacking.^2^ Therefore, exploring the pathogenic mechanism of severe infections and effectively preventing their occurrence are still priorities for current research. Previous studies by Chen et al. have shown that SNP in multiple host inflammation‐related genes, such as *TLR3*, *IL‐8*, *CPT2*, and *IRAK4*,^6^, ^7^, ^13^, ^14^ are associated with susceptibility to severe EV71 infection. It has been speculated that *TMEM173*, as an interferon stimulating factor and cGAMP interacting factor, may be involved in the immune, inflammatory, and cell death processes triggered by viral infections.^15^, ^16^ In this study, we aimed to explore the relationship between the *TMEM173* rs7447927 locus and severe EV71 infections in children.

We found that infected patients, especially those with severe infection, had a significantly higher frequency of the G allele and GG genotype than the controls. Patients with the G allele and GG genotype showed increased EV71 infection susceptibility and severity. These results suggest that children with the *TMEM173* rs7447927 G allele and GG genotype may be susceptible to severe EV71 infection in the Chinese population. Combined with the SNP sequencing results, it was predicted that the rs7447927 nucleotide polymorphism is in the third exon region of the *TMEM173* gene, which is a synonymous mutation. In rs7447927, the C allele becomes a G allele, which does not change the amino acid sequence of the protein but may influence protein activity. The effect of this change on protein activity has been shown in previous studies, revealing that rs7447927 has similar results as telomere shortening.^17^

WBC, CRP levels, and fever duration in children with the GG genotype were significantly higher than those in children with other genotypes, both in the infected and severely infected groups. As reported previously, higher WBC count, CRP level, and longer fever duration are markers of severe EV71 infection.^18^ This further verifies that the GG genotype may be involved in the process of severe infection. In addition, a higher BG concentration was found in patients with the GG genotype in the group with severe cases, which may reflect a more severe infection or complications.^19^ Combined with the significant increase in the AST and ALT indices (which represent liver function), frequency of vomiting, spirit changes, and EEG abnormalities (representing nervous system symptoms) in children with the GG genotype, we can further confirm that the GG genotype is involved in the process of severe EV71 infections. Unlike previous studies that showed that boys under 4 years of age were prone to severe infection, there were no significant differences in gender or age among children with different genotypes in this study.^19^ This may be related to the fact that this study was conducted only on Asian Han children.

TMEM173, which is located between the endoplasmic reticulum and Golgi, is a key adaptor protein for macrophages or monocytes to promote the production of type 1 interferon for immune response.^11^ After TMEM173 binds to cGAMP, TMEM173 is stimulated, which then transfers to the Golgi and activates transcription factor IRF3 by recruiting phosphokinase tank binding kinase 1 (TBK1).^11^ Subsequently, the active IRF3 transfer nucleus induces the transcription of the type I IFN gene, which is involved in infection and antitumor immune responses.^11^ In our study, we found that the IFN‐α levels in the severely infected group were significantly higher than those in the mildly infected group, and the serum IFN‐α levels of patients with the GG genotype in the severely infected group were significantly higher than those of patients with the CC and GC genotypes. No significant differences were observed in mild EV71 disease in children. This proved that *TMEM173* rs7447927 affects the development of EV71 infection by altering the expression of IFN‐α.

The actual mechanism of *TMEM173* rs7447927 in terms of promoting EV71 infection severity is unclear, but the following hypotheses may be credible. First, after EV71 infection, its specific antigen (RNA) is recognized by the surface or internal receptors (Toll‐like receptor, NOD‐like receptor, RIG receptor, etc.) of the innate immune cells (such as macrophages). Through the transmission of the intracellular signal molecule TMEM173, the IRF3/7 transcription factor is activated to initiate the expression of type I interferon, playing an antiviral role.^11^, ^20^ Second, the *TMEM173* rs7447927 G allele and GG genotype may cause the abnormal activation of TMEM173 during EV71 infection, resulting in inflammation and an imbalance of the immune network, leading to severe infection. Third, rs7447927 may influence TMEM173 activity and further affect the recruitment of TGF‐β‐activated kinase 1 and IκB kinase, thereby activating the production of NF‐κB‐mediated inflammatory cytokines, such as TNF‐α, IL‐6, and CXCL10. Finally, the rs7447927 mutation affects TMEM173 activity and promotes autophagy and cell death (including apoptosis and scorch death). Apoptosis of lymphocytes and dendritic cells releases a large number of DAMPs (including HMGB1, cfDNA, and histone), which leads to cytokine storm, immunosuppression, and coagulation activation, and ultimately ending with multiple organ failure (such as liver, heart, and brain).^21^, ^22^ The pathogenic mechanism of EV71 is related to virulence, host genetic background, and other factors. In this study, the relationship between the severity of EV71 infection and the genetic background of the host was discussed, which will help clinicians to understand severe EV71 infections from a genetic perspective, further explore its pathogenesis, and provide a theoretical basis for effective prevention and early intervention.

This study had some limitations. First, the samples collected were not sufficient to represent the whole population; therefore, the characteristics of the study sample only reflected a portion of the entire population. Second, other cytokines and inflammatory factors in the serum related to immune disorders caused by EV71 infection were not detected or analysed. Lastly, this study did not genotype or sequence the EV71 virus, but C4 was the most popular virus type in this period according to other reports.^23^, ^24^, ^25^

## AUTHOR CONTRIBUTIONS

**Jie Song**: Data curation, writing – original draft, visualization, investigation. **Yedan Liu**: Data curation, methodology, software, writing – review & editing. **Ya Guo**: Validation, formal analysis, data curation, writing – review & editing. **Peipei Liu**: Validation, data curation, visualization. **Fei Li**: Validation, resources, data curation. **Chengqing Yang**: Validation, investigation, resources. **Fan Fan**: Validation, writing – review & editing. **Zongbo Chen**: Validation, writing – review & editing, supervision, project administration, funding acquisition. All authors contributed to the writing of the final manuscript. All members of our Study Team contributed to the management or administration of the trial.

## CONFLICT OF INTEREST

The authors declare no conflict of interest.

## ETHICS STATEMENT

All the procedures in this study were in accordance with the standards of the Ethics Review Committee of the Affiliated Hospital of Qingdao University. We obtained informed consent from the children's parents.

## Data Availability

Some or all data, models, or code generated or used during the study are available from the corresponding author by request.
